# EHItk: a toolkit for accessing Earth Hologenome Initiative data resources

**DOI:** 10.1093/bioadv/vbag199

**Published:** 2026-07-15

**Authors:** Antton Alberdi, Garazi Martin-Bideguren, Jonas Lauritsen, Nanna Gaun, Elsa Brenner, Lucas Padilha, Amalia Bogri, Ostaizka Aizpurua

**Affiliations:** Center for Evolutionary Hologenomics, Globe Institute, University of Copenhagen, Copenhagen, 1350, Denmark; Center for Evolutionary Hologenomics, Globe Institute, University of Copenhagen, Copenhagen, 1350, Denmark; Center for Evolutionary Hologenomics, Globe Institute, University of Copenhagen, Copenhagen, 1350, Denmark; Center for Evolutionary Hologenomics, Globe Institute, University of Copenhagen, Copenhagen, 1350, Denmark; Center for Evolutionary Hologenomics, Globe Institute, University of Copenhagen, Copenhagen, 1350, Denmark; Center for Evolutionary Hologenomics, Globe Institute, University of Copenhagen, Copenhagen, 1350, Denmark; Center for Evolutionary Hologenomics, Globe Institute, University of Copenhagen, Copenhagen, 1350, Denmark; Center for Evolutionary Hologenomics, Globe Institute, University of Copenhagen, Copenhagen, 1350, Denmark

## Abstract

**Motivation:**

The Earth Hologenome Initiative (EHI) is generating standardized datasets that jointly capture host genomic and microbial metagenomic—namely hologenomic—information across wild vertebrates. These resources include thousands of shotgun hologenomic datasets and metagenome-assembled genomes (MAGs), accompanied by extensive metadata describing host biology, sampling context, and sequencing procedures. Although these datasets are made publicly available, efficient access to them remains challenging due to the distribution of data across multiple repositories and the complexity of the associated metadata.

**Results:**

We present EHItk, a lightweight Python package and command-line toolkit that enables programmatic discovery and retrieval of EHI datasets and their metadata. EHItk allows users to query hologenomes and MAGs using biologically meaningful metadata filters. The software translates these filters into SQL queries against a local database and supports downloading matched raw FASTQ reads and genome FASTA files. By simplifying metadata-driven dataset discovery and retrieval, EHItk facilitates the integration of EHI resources into bioinformatic pipelines and enables large-scale comparative analyses across hosts and microbial genomes.

**Availability and implementation:**

EHItk supports Python 3.10 and later. It is distributed as open-source software under the GNU General Public License v3 and is available from PyPI, Bioconda and GitHub: https://github.com/earthhologenome/ehitk

## 1 Introduction

The study of host-microbiota interactions increasingly relies on hologenomic datasets, in which host genomic and microbial metagenomic information, hereafter referred to as hologenomic information, are analysed jointly (Lan *et al.* 2021, Cheng *et al.* 2023, Guo *et al.* 2023, Tock *et al.* 2025, Wei *et al.* 2025). This integrative perspective enables researchers to investigate ecological and evolutionary processes across biological scales, from microbial community composition to host population genetics (Alberdi *et al.* 2022).

The Earth Hologenome Initiative (EHI) was established to generate and analyse standardized hologenomic data across wild vertebrates worldwide (Leonard *et al.* 2024). Using harmonized sampling protocols, sequencing workflows, and bioinformatic pipelines, the initiative produces shotgun sequencing datasets that capture DNA from both vertebrate hosts and associated microbial communities (Lauritsen *et al.* 2025, Pietroni *et al.* 2025). The raw sequencing data, as well as the metagenome-assembled genomes (MAGs) derived from them, are archived in public repositories, and published in data releases that provide the context of the generated resources (Gaun *et al.* 2025). Each dataset is accompanied by rich metadata describing host taxonomy, sampling context, geography, and laboratory processing, which enable researchers to perform comparative analyses across taxa, environments, and microbial lineages.

Although tools like NCBI Entrez utilities (Sayers *et al.* 2010) or European Nucleotide Archive (ENA) APIs (David *et al.* 2026) allow researchers to automate data retrieval within analysis pipelines, the practical use of such datasets often requires significant manual effort. As many public repositories lack the capability to store and display all the metadata in a user friendly way, researchers must identify relevant samples by inspecting metadata tables stored elsewhere, determine the location of the archived files, and then download large volumes of data using multiple software and strategies (Hughes *et al.* 2023). To address this gap, project-specific programmatic interfaces and command-line utilities are becoming more common, as they enable context-rich, scalable access to biological datasets (Khoroshevskyi *et al.* 2023, LeRoy *et al.* 2024, Rogers *et al.* 2025).

Here we introduce EHItk, the Earth Hologenome Initiative Toolkit, a command-line application designed to facilitate the discovery and retrieval of EHI data and metadata. EHItk provides a simple query interface for filtering datasets by host metadata, microbial taxonomy, and genome quality metrics, while also enabling direct download of sequencing data and genome assemblies. The toolkit bridges the gap between the EHI database and downstream bioinformatic analyses, allowing researchers to seamlessly integrate EHI data into computational workflows.

## 2 Implementation

### 2.1 Architecture

EHItk is implemented in Python 3.10+ and designed as both a lightweight command-line interface (CLI) and a Python package for programmatic access to EHI data resources. The software interacts with a local SQLite database containing curated metadata for EHI datasets (Gaffney *et al.* 2022). The database integrates metadata describing host taxonomy and phenotype, sampling event and environment, sequencing libraries and read files, and reconstructed MAGs and genome-quality metrics.

The modular architecture comprises four main components: a query engine, which constructs SQL queries from CLI arguments; a database interface, which manages SQLite access and metadata retrieval; a download manager, which handles file transfers and progress monitoring; and a manifest logger, which records download outcomes for reproducibility. EHItk translates command-line filters into SQL queries against the database, returning matching datasets in tabular format on screen or stored as CSV/TSV files. For data retrieval operations, the toolkit resolves download URLs stored in the database and fetches the corresponding files. Software installation can be done via PyPI.

In addition to the CLI, EHItk exposes a public Python API centred on the ‘ehitk.Database’ class, which can be used either with the bundled catalogue or with a user-supplied SQLite database. The database object acts as a context manager and provides access to ‘specimens’, ‘hologenomes’, and ‘mags’ collections. These collections expose query, values, stats, and, where applicable, fetch methods corresponding to the CLI functionality. Query operations return typed record objects, while summary and retrieval operations return structured result objects, allowing EHItk to be integrated directly into Python-based analyses and workflow-generation scripts.

### 2.2 Data resources

A key feature of the EHI is the hierarchical organization of its data resources. Data generation begins with the collection of biological samples from wild animal specimens. During sampling events, standardized metadata describing the environment (e.g. biome, country), the host specimen (e.g. species, sex, body measurements), and the collected sample (e.g. sample type) are recorded. Some samples, such as blood or tissue, are primarily used for generating host genomic data, whereas others, typically faecal samples, are used for metagenomic sequencing. Metagenomic datasets are subsequently assembled to reconstruct MAGs. A detailed description of the EHI data generation workflow is provided in Leonard *et al*. (2024).

EHItk represents this structure through three primary resource types: specimens, hologenomes, and MAGs. The specimens resource contains metadata describing host individuals and the environmental context of sampling events. The hologenomes resource includes metadata associated with sequenced samples, encompassing all sample types from which shotgun sequencing data were generated. Hologenome records also include URLs linking to sequencing data archived in the ENA, enabling programmatic retrieval. The MAGs resource contains metadata describing bacterial and archaeal genomes reconstructed from metagenomic assemblies, together with links to their corresponding FASTA files, which can likewise be accessed programmatically. The three resource levels are interlinked through index identifiers: MAG records include the identifier of the hologenome from which they were reconstructed, while hologenome records contain the identifier of the specimen from which the biological sample was obtained.

### 2.3 Command line interface

**Figure 1:**
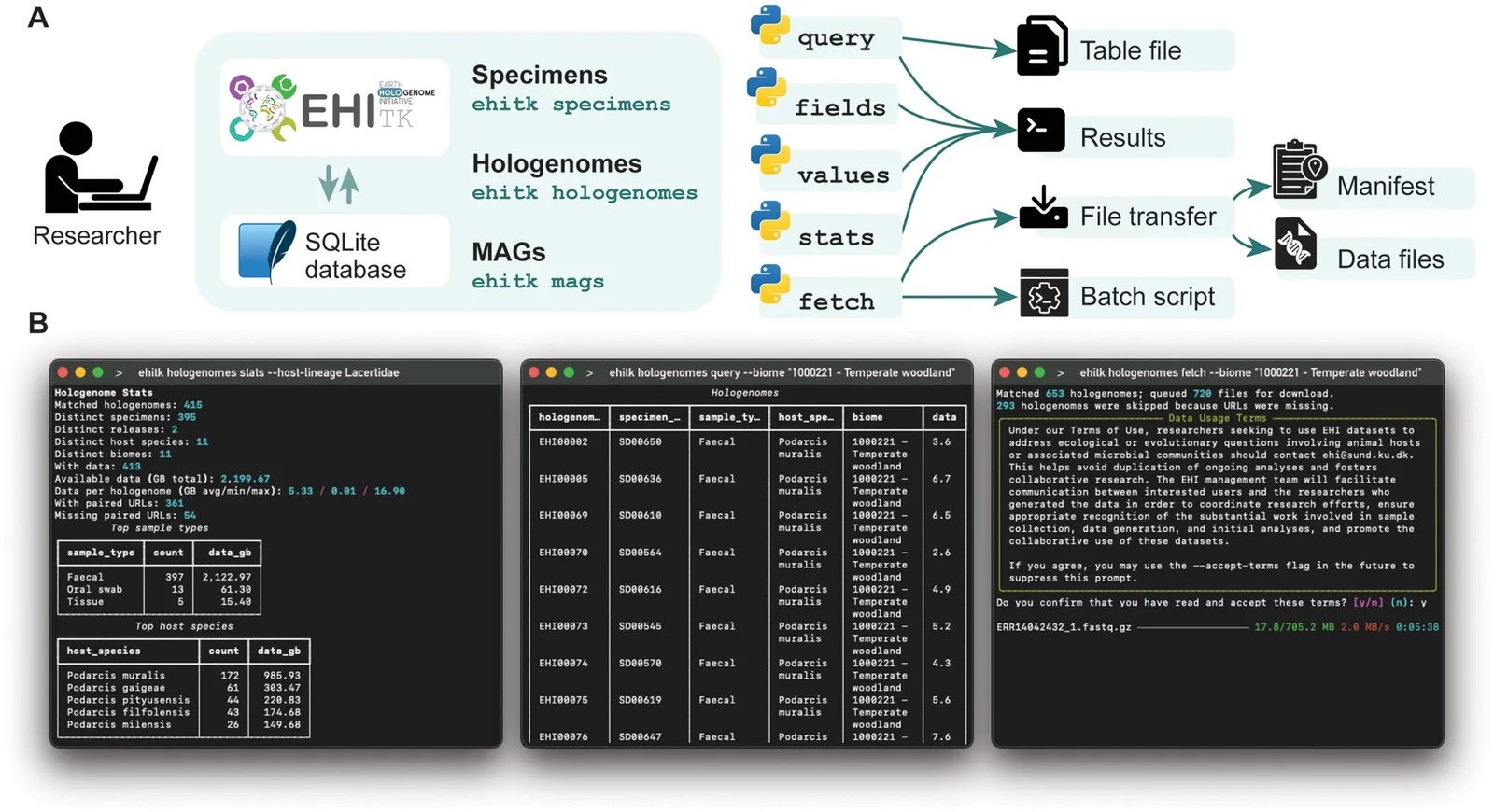
Overview of EHItk. (A) Architecture and workflow of the software. (B) Screenshots of example commands and their command line outputs.

EHItk is organized as a noun–verb CLI rooted at ‘ehitk’. The top level defines the data domain to be analysed, namely ‘specimens’, ‘hologenomes’, or ‘mags’, and each domain then exposes a small, consistent set of actions (Fig. 1). Specifically, ‘query’ returns matching records, ‘fields’ lists valid field names, ‘values’ reports distinct values and their frequencies for a selected field, ‘stats’ summarizes the filtered subset, and ‘fetch’ resolves and retrieves associated sequence files. This design gives the toolkit a uniform structure across biological entities while preserving entity-specific functionality; e.g. ‘fetch’ is implemented for hologenomes and MAGs, whereas specimen commands are limited to metadata exploration. Because EHItk operates entirely through the command line, it can be integrated into reproducible pipelines using workflow systems such as Snakemake or Nextflow (Köster and Rahmann 2012, Di Tommaso *et al.* 2017). Commands that emit rectangular data can display rich tables interactively or write CSV/TSV output either to files or to standard output for shell piping.

### 2.4 Metadata filtering

EHItk provides domain-specific filters for the three core data entities—specimens, hologenomes, and MAGs—reflecting common biological and ecological research questions. Specimens can be filtered using host-related metadata such as taxonomic identifier, species, lineage, sex, and morphological measurements, including weight and length. Hologenomes can be filtered using attributes such as host taxonomy (e.g. species, lineage, taxonomic identifier), sample type, biome, country, geographic coordinates, and associated specimen traits. MAGs can be filtered using genome-related attributes such as microbial taxonomy, inferred quality class, and parent hologenome identifier, as well as host-associated metadata including host taxonomy, geography, and specimen measurements.

Because the underlying SQLite tables are relationally linked, higher level metadata can be propagated to downstream resources. For example, hologenomes can be filtered using specimen-derived attributes, and MAGs can be filtered not only by genome-level properties but also by metadata inherited from their associated hologenomes and specimens. For hierarchical identifiers, EHItk supports descendant-aware filtering. Environment Ontology (ENVO) biome filters can expand parent biome terms to the descendant terms represented in the catalogue, and NCBI host-taxid filters can similarly expand higher-level host taxa to descendant host taxa. These descendant maps are bundled with the package for offline and reproducible queries. This relational structure enables users to query any resource using filters defined either at its own level or at upstream levels in the data model.

Across modules, EHItk also supports identifier-based filtering, range-based filtering for numeric fields, and comma-separated multi-value filters for selecting multiple categories within a single command. For advanced use cases, EHItk additionally supports custom SQL predicates through the ‘--where’ argument.

### 2.4 Data retrieval

EHItk includes commands for downloading matched datasets directly from remote archives. Hologenome downloads retrieve paired-end sequencing reads, while MAG downloads retrieve genome FASTA files. The software includes progress monitoring of the download processes and, to ensure reproducibility, records each download attempt in a JSON Lines manifest that contains the timestamp, dataset identifier, download URL, file checksum and transfer status. This manifest enables users to track dataset provenance and resume incomplete downloads.

The active catalogue can be inspected with the EHItk database command, which reports whether the database is bundled or user supplied, together with its path, size, and SHA256 checksum. Database releases are distributed as standalone versioned SQLite artifacts in addition to the catalogue bundled with the Python package. Users can therefore pin analyses to a specific catalogue version using the ‘- -db’ command-line option or the ‘ehitk.Database(“/path/to/database.sqlite”)’ Python API. Updated catalogues will be released with future software and data releases, and older catalogue versions will remain available so analyses can be reproduced by selecting a specific database artifact.

## 3 Use cases

### 3.1 Comparative analyses of microbiomes across host lineages

Host evolutionary history is one of the primary factors shaping the structure and function of host-associated microbiomes (Groussin *et al.* 2017). Comparative analyses across vertebrate clades can reveal patterns of microbial diversification and adaptation linked to host ecology, diet, or physiology (Ley *et al.* 2008, Song *et al.* 2020). Using EHItk, a researcher interested in comparing reptile-associated microbiomes to those of mammals could rapidly identify and retrieve metagenomes associated with particular host lineages. For example, they could first explore the statistics and query the catalogue for faecal metagenomes derived from reptiles and mammals. After identifying relevant records, the corresponding sequencing data could be downloaded.

*ehitk hologenomes stats- -host-lineage Reptilia*

*ehitk hologenomes stats- -host-lineage Lacertidae*

*ehitk hologenomes stats- -host-lineage Mammalia*

*ehitk hologenomes stats- -host-lineage Rodentia*

*ehitk hologenomes query- -host-lineage Lacertidae- -sample-type faecal*

*ehitk hologenomes fetch- -host-lineage Lacertidae- -sample-type faecal*

*ehitk hologenomes query- -host-lineage Rodentia- -sample-type faecal*

*ehitk hologenomes fetch- -host-lineage Rodentia- -sample-type faecal*

### 3.2 Exploring geographic patterns in host-associated microbiomes

Environmental conditions such as climate, habitat type, and geographic location can strongly influence microbial community composition (Kim *et al.* 2021, Andreu-Sánchez *et al.* 2025). Researchers studying large-scale ecological patterns may therefore wish to retrieve datasets associated with specific geographic regions or environmental contexts. A typical workflow would begin with exploring the available metadata categories in the database. For example, a researcher interested in microbiomes from woodland ecosystems can first list all biome categories present in the database to identify relevant environmental classifications. Once the desired biome category is identified, summary statistics can be obtained to assess the number of available hologenomes and host taxa represented. These statistics allow researchers to evaluate whether sufficient data are available for robust comparative analyses before initiating downloads. After identifying the relevant datasets, the researcher can query the hologenome catalogue to inspect the corresponding records and subsequently download the sequencing data for downstream analysis.

*ehitk hologenomes fields*

*ehitk hologenomes values- -field biome_name*

*ehitk hologenomes stats- -biome-name “Temperate woodland”*

*ehitk hologenomes query- -biome-envo-id ENVO: 01000221*

*ehitk hologenomes fetch- -biome-envo-id ENVO: 01000221*

### 3.3 Targeted retrieval of microbial genomes for comparative genomics

Genome-resolved metagenomics provides access to thousands of microbial genomes from host-associated communities. Researchers interested in specific microbial taxa often need to retrieve genomes belonging to a particular lineage across multiple hosts and environments. EHItk facilitates this process through taxonomy-based filtering of the MAG catalogue. For example, a researcher studying the genus Escherichia could query the database to identify all MAGs assigned to this lineage and subsequently restrict the selection to genomes that meet defined quality thresholds. The resulting genomes can then be downloaded for phylogenomic analysis or functional annotation. In addition, the corresponding metagenomes can be retrieved to provide ecological context for the reconstructed genomes.

*ehitk mags query- -genus Escherichia*

*ehitk mags query- -genus Escherichia\*

*- -where “completeness >= 95 AND contamination <= 2”*

*ehitk mags fetch- -genus Escherichia\*

*- -where “completeness >= 95 AND contamination <= 2”*

## 4 Discussion

The volume and diversity of hologenomic datasets are rapidly increasing as endeavours such as the EHI expand their sampling across host taxa and geographic regions (Leonard *et al.* 2024). The first EHI data release already contains hundreds of sequencing libraries and tens of thousands of microbial genomes (Gaun *et al.* 2025), and future releases are expected to substantially expand this catalogue. Efficient tools for discovering and retrieving these datasets are therefore essential. EHItk addresses this need by providing a lightweight programmatic interface that bridges the EHI metadata database and downstream analyses. Compared with manual download workflows, EHItk offers several advantages: metadata-driven dataset discovery, automated retrieval of sequencing and genome files, reproducible download manifests, and seamless integration with bioinformatic pipelines.

By simplifying access to EHI datasets, EHItk lowers the barrier for comparative analyses of host–microbiota systems across taxa and environments. This contributes to the broader goal of the EHI to establish high-quality, open hologenomic research on wild animals and their associated microorganisms. At the same time, users are expected to adhere to the EHI data usage guidelines, which aim to ensure appropriate credit to researchers who invested substantial effort in sample collection, data generation, and initial analyses.

As stated in the usage terms in the data release publications (Gaun *et al.* 2025), researchers wishing to use EHI datasets to investigate ecological or evolutionary questions involving animal hosts or associated microbial communities should first contact the corresponding author listed in the data release or this manuscript. This practice helps avoid duplication of ongoing analyses and encourages collaborative research. Following initial contact, the EHI management team facilitates communication between interested users and the researchers who generated the data to co-ordinate research efforts and promote collaborative use of the datasets. The use cases presented above illustrate research directions that would require prior communication under these guidelines.

As the EHI continues to expand its global sampling efforts, tools such as EHItk will be essential for ensuring that these datasets remain accessible, discoverable, and readily usable by the broader research community under a collaborative and respectful approach.

## Data Availability

EHItk can be downloaded and used as a python package from Python Package Index (PyPI): https://pypi.org/project/ehitk. The development repository is hosted in Github: https://github.com/earthhologenome/ehitk. Snapshots of software and database releases are archived in Zenodo under the digital object identifiers (doi) 10.5281/zenodo.20989016 and 10.5281/zenodo.20430293, respectively. The software is released under GNU General Public License v3.
